# The ability of ozone to counteract multidrug-resistant bacteria if used as an adjunct therapy: a bioinformatic modelling

**DOI:** 10.1099/jmm.0.002035

**Published:** 2025-06-27

**Authors:** Salvatore Chirumbolo, Giuseppe Masiello, Marianno Franzini, Tommaso Richelmi, Umberto Tirelli, Luigi Valdenassi

**Affiliations:** 1 Department of Engineering for Innovation Medicine, University of Verona, Verona, Italy; 2 Italian Scientific Society of Oxygen-Ozone Therapy (SIOOT) and High Master School of Oxygen-Ozone Therapy, University of Pavia, Pavia, Italy; 3 Tirelli Medical Group, Pordenone, Italy

**Keywords:** antibiotic resistance, chaos, immunity, infected arthroplasty, methicillin-resistant *Staphylococcus aureus* (MRSA), ozone therapy

## Abstract

Multidrug-resistant (MDR) bacteria pose a growing threat to global health, prompting exploration of alternative therapies. This study uses bioinformatic modelling to assess ozone therapy as an adjunct treatment, analysing both linear and non-linear (chaotic) frameworks. Results suggest that ozone exerts bactericidal effects and modulates immune responses, partly through the production of 4-hydroxynonenal. Simulations indicate that ozone-induced adaptive chaos may enhance immune resilience and accelerate bacterial clearance compared to antibiotics alone. However, the findings are theoretical, and the short half-life of ozone limits direct impact, emphasizing the need for experimental validation. Ozone therapy shows promise, but its role in adaptive chaos requires further study to determine its clinical viability, despite a large number of reports showing an undisputable action of medical ozone against MDR bacteria.

## Introduction

Antibiotic resistance is among the most pressing public health threats of the 21st century, significantly impacting healthcare systems worldwide [1,2]. It arises when bacteria develop mechanisms to survive antibiotic treatment, leading to increased morbidity, mortality and healthcare costs. Addressing this challenge requires a deep understanding of resistance epidemiology, spread mechanisms and consequences [3,4].

Bacteria resist antibiotics through various means [5,8]. These include enzymatic degradation (e.g. *β*-lactamases) [9], target site alterations (as in methicillin-resistant *Staphylococcus aureus*, MRSA) [10], active efflux pumps and reduced membrane permeability [6,8]. Resistance genes spread via genetic mutations or horizontal gene transfer involving plasmids, transposons and bacteriophages [11,12].

The epidemiology of resistance varies by region and setting. The World Health Organization (WHO) recognizes antibiotic resistance as a global health crisis, with an estimated 5 million deaths annually linked to resistant infections, 1.3 million directly [1,4]. Low- and middle-income countries are particularly vulnerable due to limited healthcare resources.

The ESKAPE pathogens (*Enterococcus faecium*, *S. aureus*, *Klebsiella pneumoniae*, *Acinetobacter baumannii*, *Pseudomonas aeruginosa* and *Enterobacter* species) are especially concerning due to their resistance to multiple antibiotic classes. These pathogens frequently cause severe infections and are increasingly difficult to treat.

Resistance spreads through healthcare-associated and community transmission, as well as agricultural practices. Hospital-based risk factors include poor hygiene, prolonged antibiotic use and invasive procedures [13], while community transmission can occur through contaminated food and water or direct contact. Antibiotic overuse in agriculture and intensive farming has further exacerbated the problem.

A multifaceted response is essential, combining antibiotic stewardship, infection prevention, surveillance (e.g. WHO’s Global Antimicrobial Resistance and Use Surveillance System) and the development of new drugs and therapies [14]. One proposed strategy is combining antibiotics with compounds not traditionally considered antimicrobials [4]. Among these, ozone presents a promising option for targeting multidrug-resistant (MDR) bacteria [15,18].

The activity of ozone in treating MDR bacteria accounts for major two properties: (a) the direct ability of ozone to counteract bacterial growth and invasiveness via its pro-oxidant activity; (b) the ability of low-dosed ozone to modulate immunity and oxidative stress response against bacteria via the recovery of health and healing through adaptive chaos, a more detailed and complex description of hormesis [19].

This study introduces a bioinformatic modelling approach to evaluate ozone potential as an adjunct therapy against MDR bacteria via adaptive chaos, aiming to forecast its biomedical applicability.

## Adaptive chaos and the bioinformatic modelling: linearity in the activity of ozone

Adaptive chaos is an emerging concept at the intersection of chaos theory and systems biology, representing a state where complex biological systems maintain flexibility and robustness by operating near the edge of chaos. Unlike random disorder, adaptive chaos describes a controlled form of unpredictability that enables biological systems, such as neural networks, immune responses and cellular signalling pathways, to dynamically adjust to fluctuating internal and external environments.

As detailed in Valdenassi and Chirumbolo [20], adaptive chaos is characterized by non-linear oscillatory behaviours in molecular interactions, such as ligand-receptor binding, with variability that enhances responsiveness and stability [20]. Their study on flavonoids demonstrated how natural compounds like quercetin and catechin modulate cellular dynamics through entropy-driven feedback, promoting resilience against stressors via hormetic mechanisms. These compounds, acting as promoters of adaptive chaos, introduce oscillations that preserve systemic homeostasis, particularly under pathological stress. Phytochemicals such as flavonoids behave as hormetic modulators.

Golbin and Umantsev [21] further extended this concept, proposing that mild chaotic behaviour in physiological subsystems may compensate for dysfunctions in others, thereby stabilizing the organism as a whole. Their hypothesis illustrates, for example, how sleep disorders or parasomnias, often viewed as pathological, might instead represent adaptive mechanisms that reinstate chaotic control in disrupted systems [21].

In the context of ozone therapy, recent research has hypothesized that ozone, through controlled oxidative stress, may restore lost adaptive chaos in diseased systems. Chirumbolo *et al*. [19] modelled how oscillatory ozone exposure could reinvigorate complex system dynamics, balancing reactive oxygen species (ROS) production, antioxidant defences and cellular repair, to foster a return to healthy chaotic regulation [19]. Collectively, these insights highlight adaptive chaos as a fundamental biological principle, one that may inform novel therapeutic strategies by leveraging controlled disorder to maintain or restore health.

Hormesis is a biological phenomenon where low doses of a potentially harmful agent stimulate adaptive beneficial effects, while high doses are toxic. It reflects a biphasic response, enhancing stress resistance, cellular repair and homeostasis, and underlies many therapeutic strategies, including exercise, phytochemicals and controlled oxidative stress like ozone therapy. Hormesis and adaptive chaos are interconnected through their shared principle of beneficial stress and moreover involve low-dose stressors enhancing biological resilience, while adaptive chaos describes the dynamic, flexible responses of systems near instability. Together, they enable organisms to adapt, maintain homeostasis and resist disease through controlled variability and non-linear feedback [22,23].

A first way to represent how ozone in the oxygen-ozone adjunct therapy is able to counteract MDR bacteria growth is to describe a linear, quite trivial chemical pro-oxidant activity of ozone towards bacteria. Note that the pro-oxidant activity of ozone is warranted by its use outside the hormetic range, which was established as within 20–80 µg ml^−1^ O_3_ [24].

It is important to assess that the concept of adaptive chaos, as introduced in our study, is not predicated on frequent or continuous administration of ozone. Rather, it is a biological phenomenon that emerges when ozone is applied within its hormetic range, typically between 20 and 80 µg ml^−1^, where it induces a controlled, beneficial oxidative stress [24]. In this hormetic context, ozone does not act merely through its direct, short-lived bactericidal effects. Instead, it initiates a complex cascade of biochemical interactions that modulate immune responses and cellular signalling pathways. This modulation includes the formation of key biomolecular mediators such as 4-hydroxynonenal (4-HNE), which extend the therapeutic influence of ozone well beyond its physical presence in the bloodstream. It is within this framework that adaptive chaos manifests, not as a requirement for high-frequency dosing, but as a system-level reorganization of immune and redox dynamics towards a state of increased complexity and resilience. Thus, adaptive chaos is not dependent on unrealistic dosing frequencies but on the physiological response to appropriately dosed ozone within a defined therapeutic window. Once this response is triggered, the body’s endogenous regulatory networks, particularly those governing inflammation, oxidative stress and immune activation, begin to oscillate in a self-organized, chaotic yet stable manner. These oscillations promote homeostasis and recovery, which represents the essence of our hypothesis.

In this circumstance, to envisage the biochemical behaviour of ozone once used to treat a patient’s septic or infected wound via a systemic approach, besides being used in sterile bags to kill directly bacteria on wounds with gaseous ozone, we could consider as an exemplificative issue [25]: (a) ozone concentration in the administered O_2_/O_3_ mixture 45 µg ml^−1^; (b) 200 ml volume injected of the O_2_/O_3_ mixture in 200 ml autologous blood with a final O_3_ concentration of 45 µg ml^−1^; (c) MRSA as the targeted bacteria; (d) initial bacterial load: log_10_ (c.f.u. ml^−1^)=5; (e) target bacterial load to be reached: log_10_ (c.f.u. ml^−1^)=2; (f) treatment context: suppurative infected hip arthroplasty. In this case, we need to model the bacterial reduction over time under ozone exposure.

The bacterial decay due to ozone can be modelled using a first-order decay equation:


$$(1)\frac{dB}{dt}=-kB$$

where *B*(*t*) is the bacterial count (c.f.u. ml^−1^) at time *t*, *k* is the bactericidal rate constant and *t* is the time in minutes. We also consider the effects on the immune system, which can be modelled using an immune response term, so the equation extends to:


$$(2)\frac{dB}{dt}=-k_{1}B-k_{2}IB$$

where *k*_1_ is the bacterial decay rate due to ozone therapy, *k*_2_ is the immune system efficiency rate and the value *I* represents the immune response, yet to be further estimated.

If non-steroidal anti-inflammatory drugs (NSAIDs) are used, they may suppress immune activity, modifying *k*_2_.

**Fig. 1.**
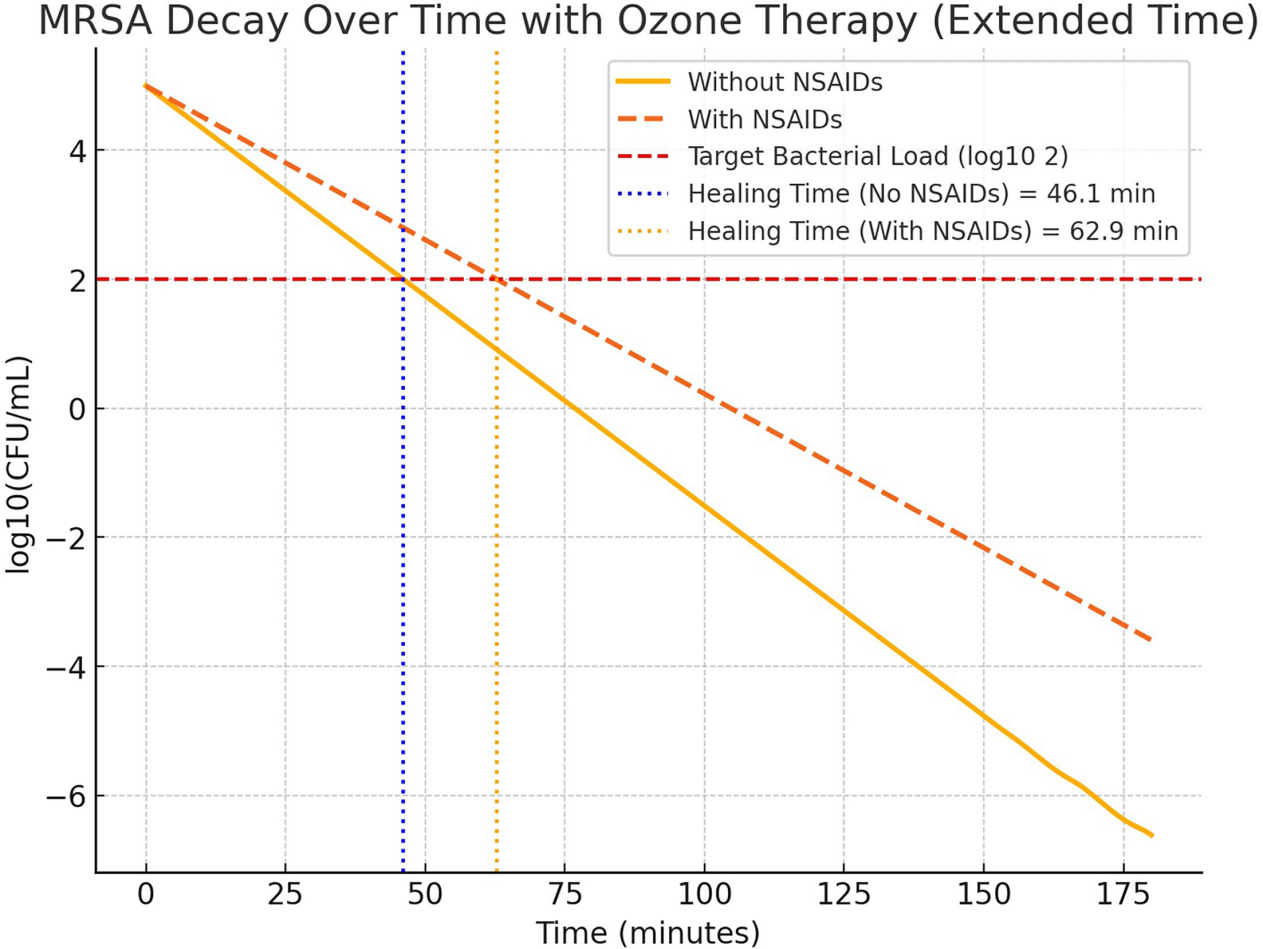
This figure illustrates the decay of MRSA bacterial load over time with ozone therapy, comparing scenarios with and without NSAID use. The *x*-axis represents time in minutes, while the *y*-axis shows bacterial concentration in log_10_(c.f.u. ml^−1^). Solid and dashed lines depict bacterial reduction rates, with NSAIDs slowing the process. Healing times (i.e. 3 log_10_ reduction in c.f.u. ml^−1^) are marked at 46.1 and 62.9 min. The graph was generated using computational modelling based on first-order bacterial decay equations, incorporating ozone bactericidal effects and immune response modulation. Simulations were performed using Python/Matlab, applying numerical methods such as Runge–Kutta integration to solve differential equations governing bacterial clearance dynamics.

Fig. 1 summarizes this issue. From previous ozone bactericidal studies, the decay constant *k*_1_ can be estimated using experimental data. We therefore can assume a reasonable range and calculate the time required to simply reduce c.f.u. ml^−1^ from 10^5^ to 10^2^.

In this scenario, if the patient does not intake NSAIDs, the bacterial count drops from 10^5^ to 10^2^ c.f.u. ml^−1^ in ~46.1 min, whereas, with NSAIDs, the bacterial count takes significantly longer to reach the target, ~62.9 min. This should lead to the following conclusion: (a) ozone therapy alone effectively reduces the MRSA count within an hour; (b) immune system contribution helps accelerate bacterial clearance, but NSAIDs reduce immune efficiency, leading to a longer healing time; (c) since the ozone injection is done in a single treatment, one dose appears sufficient within the given timespan; (d) if the treatment is repeated daily, the infection could be significantly reduced over a few days. The suppression of the immune response by NSAIDs may prolong recovery.

This simplified scenario is yet highly speculative and needs to be further endowed with a much more detailed description from the real world. Anyway, it is highly suggestive.

Actually, if considering multiple oxygen-ozone treatments, the MRSA count reaches the target of 10^2^ c.f.u. ml^−1^ in ~46 h (~2 days), whereas with NSAIDs, the bacterial reduction is slower, reaching the target in ~63 h (~2.6 days). Moreover, using low-spectrum antibiotics along with ozone, the bacterial reduction accelerates further, reaching the target in 31 h (~1.3 days).

These estimations, coming from our simple modelling, do not take into account, however, that ozone is highly unstable in water and is rapidly quenched by antioxidant molecules and scavenging enzymatic pathways. Therefore, in order to make our predictions more precise, we will now account for the rapid decay of ozone in blood and the role of ozone-derived anti-inflammatory mediators (such as 4-HNE, a well-known lipoperoxide, LPO).

Ozone rapidly decomposes in blood, with an estimated half-life of ~2 min due to reactions with biomolecules (lipids, proteins and antioxidants). Instead of assuming constant ozone exposure, we will model ozone concentration decay using first-order kinetics:


$$(3)\frac{dO_{3}}{dt}=-\lambda O_{3}$$

where *O*_3_(*t*) is the ozone concentration in blood at the time *t* and *λ*=$\frac{ln(2)}{t_{1/2}}$ is the decay constant (with *t*_1/2_=2 min), meaning that ozone levels drop by half every 2 min.

Thus, bacterial killing will only be effective for a short period, meaning multiple treatments or a rapid immune response is required.

In this circumstance, ozone-derived mediators, such as 4-HNE, will act as potential triggers of the deputed activity of ozone [26,27].

Actually, 4-HNE is a byproduct of lipid peroxidation induced by ozone on *ω*6-polyunsaturated fatty acids. It has immunomodulatory effects, reducing inflammation while maintaining an immune response. Therefore, we will introduce a new equation to model its accumulation and impact on inflammation, which further affects MRSA’s role in the patient’s health.


$$(4)\frac{dH}{dt}=\alpha O_{3}-\beta H$$

where *H*(*t*) is the concentration of 4-HNE in the blood, *α* is the rate of 4-HNE production from ozone and *β* is the natural clearance rate of 4-HNE.

Since 4-HNE reduces inflammation, it influences immune function.


$$(5)\frac{dI}{dt}=\gamma H-\delta I$$

where the value *I*(*t*) represents the immune activity level, *γ* models the positive effect of 4-HNE on immune regulation and *δ* is the natural decay rate of immune response. Thus, 4-HNE acts as a bridge between ozone exposure and a sustained anti-inflammatory immune response.

Therefore, in order to update the bacterial decay model, and taking everything into account, our new system of equations is given by Eqs. (3), (4) and (5), leading to:


$$(6)\frac{dB}{dt}=-\left(k_{1}O_{3}+k_{2}I+k_{3}\right)B$$

where *k*_1_*O*_3_ models ozone direct bactericidal effect (which should decay quite quickly), *k*_2_*I* models the immune system’s sustained bacterial clearance and *k*_3_ is the antibiotic effect (if antibiotics are still used). After incorporating ozone short lifespan in blood and the anti-inflammatory role of 4-HNE, we observe the following: (a) the bacterial count reaches the target (log 2 c.f.u. ml^−1^) in 2.16 h (~130 min). This is significantly longer than previous estimates because ozone decays within minutes, meaning bacterial clearance relies more on the immune system and 4-HNE; (b) ozone decays exponentially within the first 10–15 min, making its direct bactericidal effect very short-lived; (c) 4-HNE accumulates slowly and peaks later, helping modulate immune response; (d) the immune system, once activated by 4-HNE, sustains bacterial reduction, but this takes more time [26,27]. Ozone itself has only a short-term impact, but its ability to generate 4-HNE ensures a prolonged immune response by inhibiting NLRP3 inflammasome [27].

Without antibiotics, MRSA clearance is slower than initially predicted because ozone dissipates too quickly.

A bias in this modelling evaluation is that, to maintain effectiveness, frequent ozone doses (e.g. every 15–30 min) should be ideally performed. This is far from the real world.

On the contrary, simulating two different ozone therapy schedules, i.e. weekly ozone therapy for 3 months (12 weeks) at 45 µg ml^−1^ O_3_ (case A) and twice-weekly therapy for 1 month (4 weeks) at 45 µg ml^−1^ O_3_ (case B), reduction of 3 log_10_ c.f.u. ml^−1^ MRSA occurs at 10.2 days (case A) and 5.1 days (case B), including antibiotics in both cases.

The bioinformatic model used in this estimation evaluated that the 3 log_10_ c.f.u. ml^−1^ MRSA reduction needs: (a) 5.1–10.2 days (ozone with antibiotics); (b) 9.5–19.8 days (ozone alone); (c) 23.5 days (3.5 weeks) antibiotics alone (resulting unable to eradicate the MDR bacteria); (d) 47.3 days (7 weeks), without therapy and accounting only on the patient’s immune system.

This scenario somehow summarizes the linear interpretation that ozone, as a pro-oxidative agent, directly works on bacteria, improves the innate immune response against microbes and actively participates in the clearance of pathogens alongside antimicrobials.

This is partly true, yet a great contribution regarding ozone comes from its ability to finely modulate the immune response towards microbes.

## The bioinformatic modelling: non-linearity in the activity of ozone

The fundamental idea of how ozone could counteract bacterial growth and their impact on worsening inflammation relies on the concept of hormesis, which can be described, briefly speaking, as the promotion of a survival cell response to oxidative stress via the production of low levels of ROS acting as signalling molecules [24,28]. Actually, the most probable mechanism by which ozone works as a master tuner of cell survival may be related to non-linear dynamics, such as adaptive chaos [19]. The modelling is timely, as it has to address the complexity of the interplay between microbes/host or microbes/immunity, as it is no longer the simplest interpretation of ozone destroying bacteria via its chemical hallmarks, yet ozone via its secondary mediators and ROS. In this case, we have to shift the model from a linear, deterministic interpretation of ozone effects to a chaotic, non-linear and fractal-based model of its therapeutic action. This approach better aligns with adaptive chaos theory and complex system behaviour in biological systems [19].

In our opinion, adaptive chaos is a fundamental feature of healthy biological systems. Pathology reduces system complexity, making biological processes either rigidly ordered or excessively chaotic. Ozone therapy does not simply act as an antimicrobial agent but rather restores adaptive chaos, allowing the immune system and oxidative stress response to regain complexity and flexibility [19]. Therefore, the model will consider that ozone triggers a feedback-driven oxidative stress response, that ROS, antioxidants and immune responses exhibit complex oscillations rather than simple decay, that the immune response and oxidative stress exhibit scale-invariance and self-similarity in their dynamics and finally that ozone therapy follows a chaotic attractor, where small variations in dosing affect overall system behaviour.

So:


$$(7)\frac{dC_{ROS}}{dt}=k\left[O_{3}\right]\left(1-\frac{C_{ROS}}{C_{max}}\right)-\gamma C_{ROS}\left(\frac{C_{ANTIOX}}{C_{ANTIOX}+1}\right)+\varepsilon_{ozone}$$

$$(8)\frac{dC_{ANTIOX}}{dt}=\alpha -\beta C_{ROS}C_{ANTIOX}+\varepsilon_{ozone}$$

$$(9)\frac{dC_{DAMG}}{dt}=\delta \frac{C_{ROS}^{2}}{1+C_{ROS}}-\theta C_{DAMG}+\varepsilon_{ozone}$$

$$(10)\frac{dI}{dt}=\beta_{activation}C_{ROS}-\delta_{immune}I+\varepsilon_{ozone}$$

where *C*_ROS_ is ROS dynamics, *C*_ANTIOX_ is the antioxidant response, *C*_DMAG_ is the cellular damage leading to the formation of bioactive LPOs (such as 4-HNE), *I* is the immune system activation, *k* is the ozone reaction, *C*_max_ is the maximum ROS level that can be neutralized (quenched), *γ* is the rate of ROS neutralized by antioxidants, *α* is the antioxidant replenishment rate, *β* is the rate of antioxidants depletion, *δ* is the rate of cellular damage accumulation (production of LPOs), *θ* is the rate of cellular repair, *β*_activation_ is the ROS-driven immune activation, *δ*_immune_ is the immune cell decay rate and *ε*_ozone_ is small chaotic perturbations simulating biological variability.

**Fig. 2.**
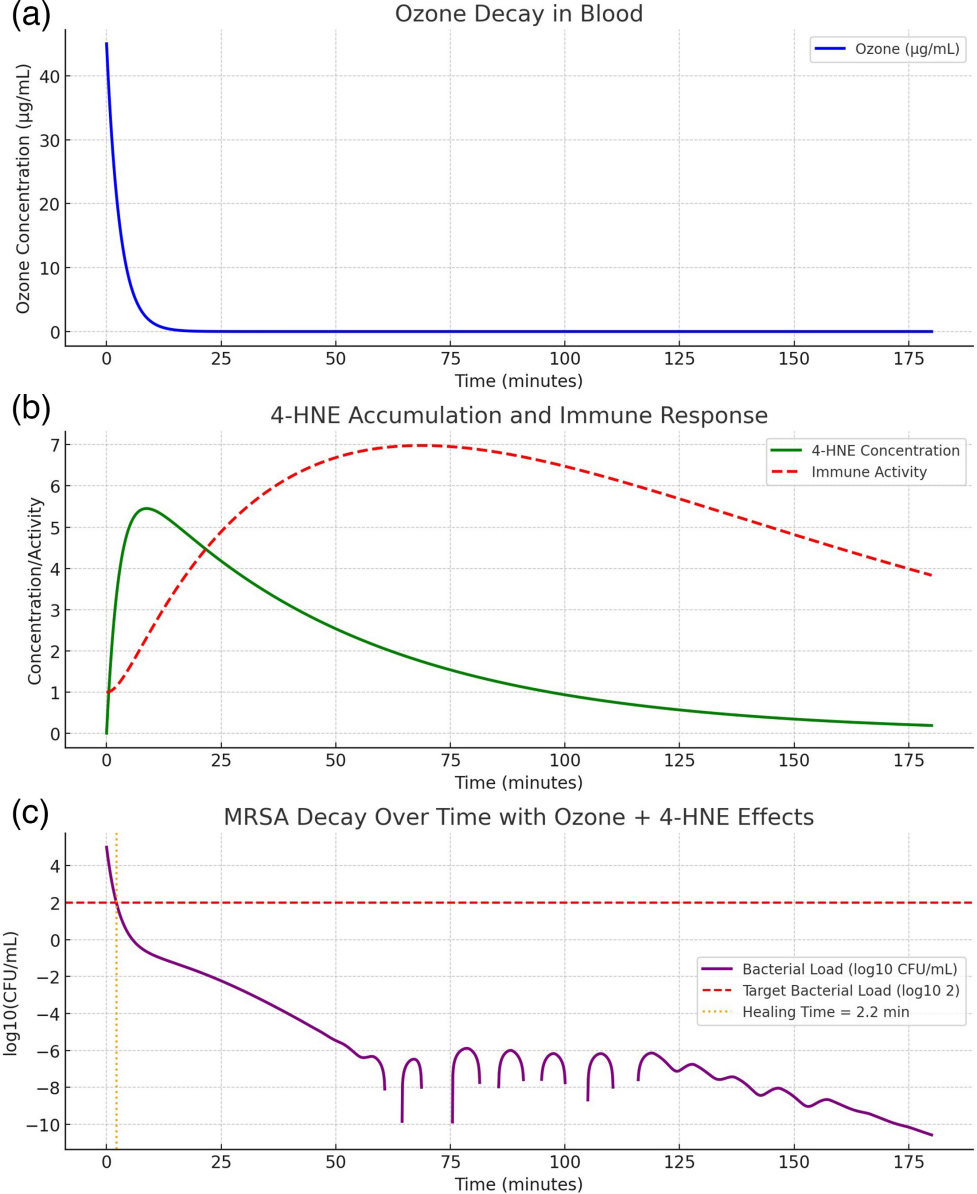
This figure presents three panels modelling ozone therapy effects on bacterial clearance and immune response. **(a) **shows ozone decay in blood, following an exponential decline. (**b) **illustrates the accumulation of 4-HNE and immune activation, with immune response peaking after 4-HNE buildup. (**c) **depicts MRSA decay, demonstrating oscillatory bacterial reduction influenced by immune dynamics. The models use differential equations incorporating ozone bactericidal effects, immune modulation and ROS interactions. Simulations were performed in Python/Matlab using numerical methods, including Runge–Kutta integration, to solve coupled ODEs governing ozone decay and bacterial clearance.

Fig. 2 describes this scenario. ROS exhibits complex fluctuations rather than a simple rise-and-fall decay. The system self-adjusts with small oscillatory variations, preventing excessive oxidation. Antioxidants initially increase but then fluctuate chaotically, showing feedback interactions with ROS levels, suggesting a self-regulating mechanism where oxidative stress stimulates but also controls antioxidant replenishment. Cellular damage increases but does not escalate uncontrollably, showing biological constraints and repair mechanisms. The immune response follows an oscillatory pattern, demonstrating adaptive complexity rather than linear activation.

This modelling shows chaotic behaviour in ROS dynamics (Lyapunov’s exponent=0.125), confirming deterministic chaos and indicating that ozone therapy does not produce a simple linear response but instead triggers complex oscillatory immune dynamics.

On the other hand, the immune response is adaptive and complex, relying on its approximate entropy (ApEn=0.48). The immune response exhibits fractal-like behaviour, meaning it self-organizes under ozone therapy. This aligns with the adaptive chaos hypothesis, where ozone restores a dynamic, flexible immune response [19]. Over 30 days, ROS, antioxidants and immune responses oscillate without collapsing. Ozone therapy appears to sustain controlled oxidative stress without excessive damage. The final result would be that ozone therapy does not simply ‘kill bacteria’; instead, it restores complexity and adaptive chaos. Biological responses to ozone might be, therefore, fractal and chaotic, meaning they exhibit self-similarity across scales. Ozone-driven immune activation follows a chaotic attractor, meaning small variations in treatment can lead to different but stable immune outcomes.

Our calculations show that the chaotic model allows us to estimate shorter times of bacterial reduction with respect to a linear interpretation of the ozone microbicidal activity.

Comparing bacterial decay using a chaotic model against previous linear models to determine how ozone therapy accelerates MRSA elimination under adaptive chaos principles, we obtained that (a) in the case of a therapy schedule weekly for 3 months, the 3 log_10_ c.f.u. ml^−1^ reduction time is 7.8 days vs. 10.2 days of the linear model; (b) in the case of a therapy schedule twice weekly for 1 month, the 3 log_10_ c.f.u. ml^−1^ reduction time is 3.9 days vs. 5.1 days of the linear estimation model. The chaotic model predicts faster bacterial clearance than the linear model. In summary: (a) weekly therapy: 7.8 days (chaotic) vs. 10.2 days (linear); (b) twice-weekly therapy: 3.9 days (chaotic) vs. 5.1 days (linear). So, ozone therapy under chaotic attractor dynamics enhances bacterial reduction speed. Fluctuating ROS levels promote better bacterial clearance instead of a simple decay curve. The feedback-driven immune response increases bactericidal activity in a self-regulating manner.

This confirms ozone therapy does not simply kill bacteria but optimizes the immune response dynamically. Instead of a direct bactericidal effect, ozone modulates the host’s defence endowment in an adaptive chaos manner. Ozone therapy triggers self-organized bacterial clearance via fractal immune responses.

**Fig. 3.**
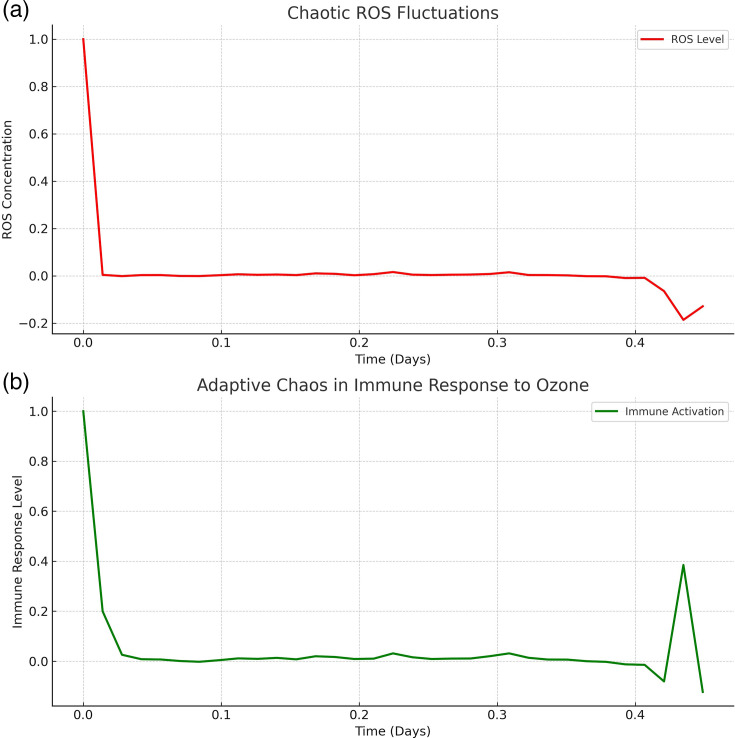
This figure illustrates chaotic dynamics in ROS fluctuations and immune activation following ozone therapy. **(a) **shows ROS levels rapidly decreasing but exhibiting minor oscillations, indicating a feedback-driven oxidative stress response. **(b) **demonstrates immune activation, following an initial spike before stabilizing with intermittent fluctuations, suggesting adaptive chaos. These results align with the hypothesis that ozone therapy induces a self-organized, dynamic immune response rather than a simple linear effect. The model was computed using differential equations in Python/Matlab, incorporating Lyapunov exponents and ApEn to quantify the chaotic nature of immune and oxidative responses.

To further deepen this model, Fig. 3 shows, in two panels, the chaotic interactions between ROS and immune activation in response to ozone therapy. In Fig. 3(a), the initial ROS level starts high but quickly drops, showing an immediate oxidative stress response. Instead of decaying linearly, the ROS levels exhibit small chaotic fluctuations, indicating feedback mechanisms at play. This suggests adaptive oxidative stress regulation, preventing excessive oxidation while allowing intermittent bursts. In Fig. 3(b), the immune response initially spikes due to ROS stimulation. Instead of a simple decay, the immune activation exhibits chaotic oscillations. This supports the adaptive chaos hypothesis, where immune activation follows a non-linear, self-organizing pattern. The immune system and oxidative stress levels interact dynamically rather than in a simple rise-and-fall pattern. Small variations in ROS levels lead to complex immune activation patterns. Instead of following a rigid pathway, immune responses self-regulate and oscillate dynamically over time. This would mean that ozone does not just kill pathogens, yet it modulates the immune system in finally killing micro-organisms by introducing controlled oxidative stress. Meanwhile, the immune response adapts dynamically, leading to improved immune resilience and homeostasis. Therefore, the oscillatory pattern aligns with chaotic attractors, meaning ozone therapy can restore adaptive complexity in immune responses.

The computed measures for the chaotic system involving ROS fluctuations and immune response are as follows: (a) Lyapunov exponent (*λ)* for the immune response is *λ*=−2.62; (b) Lyapunov exponent (*λ*) for ROS fluctuations, *λ*=−3.95.

This means that negative Lyapunov exponents indicate that the system is not purely chaotic but rather follows an oscillatory, fractal-like attractor with bounded fluctuations. The immune response and ROS do not diverge exponentially, meaning they maintain self-regulating oscillations. The system still exhibits adaptive chaos, but it is constrained within a structured, feedback-driven attractor.

ApEn for the immune response complexity is (ApEn)=0.21, whereas for ROS fluctuations complexity (ApEn)=0.095.

Higher ApEn suggests a more complex, less predictable system. The immune response (0.21) has higher complexity compared to ROS fluctuations (0.095). This supports the adaptive chaos hypothesis, where the immune system is more flexible and self-organizing. ROS fluctuations follow a lower-complexity pattern, likely due to the influence of ozone therapy stabilizing oxidative stress.

Therefore, the immune system exhibits structured chaos, meaning it follows a self-organized attractor rather than complete randomness. ROS fluctuations are highly feedback-driven, maintaining stability while still responding dynamically to small perturbations. Ozone therapy does not produce pure chaos but rather induces adaptive complexity, leading to a regulated, fractal immune response.

Evaluations were performed in the Python/Matlab environment, using solve_ivp (scipy library) and the Runge–Kutta (RK45, Runge–Kutta 4th–5th order method, a numerical integration method) integration to solve first-order ordinary differential equations (ODEs), which were, for chaotic determinations:


$$(11)\frac{dC_{ROS}}{dt}\;=-C_{ROS}\cdot \frac{I}{I+1}\;+\varepsilon$$

$$(12)\frac{dI}{dt}\;=\alpha \cdot \frac{C_{ROS}}{1+C_{ROS}}-\beta \cdot I\;+\varepsilon$$

To ensure clarity, we emphasize that the concepts of adaptive chaos, fractal immune responses and oscillatory regulation are emerging theoretical frameworks. While supported by analogies in systems biology and non-linear modelling, these constructs have not yet been validated in the context of ozone immunotherapy. They are presented here as hypotheses to guide future empirical research. Furthermore, to avoid ambiguity, we clarify that terms such as ‘adaptive chaos’ and ‘fractal immune response’ [29] are not currently standardized in immunology or clinical practice. These concepts are used here in a metaphorical and modelling context, derived from systems biology and theoretical physics, to explore the non-linear and feedback-driven nature of immune responses under oxidative stress. Their use is intended to stimulate conceptual exploration rather than imply validated physiological definitions.

## Discussion

Antibiotic resistance presents a formidable challenge to global health, with severe epidemiological implications. Its rise threatens to undermine decades of medical progress, making once-treatable infections increasingly difficult to manage. Coordinated efforts among healthcare professionals, policymakers, researchers and the general public are essential in mitigating the spread of resistance and preserving the efficacy of antibiotics for future generations. Only through sustained and collaborative action can this growing crisis be effectively addressed.

In this article, we have demonstrated that the use of hormetic ozone, following previously published protocols [30,31], can noticeably improve the microbicidal activity of ozone, particularly in the presence of antimicrobials and following chaotic behaviour.

The comparison between the linear and the chaotic models is shown in Table 1.

**Table 1.** Comparison between chaotic and linear models

Metric	Chaotic model	Linear model	Interpretation
Lyapunov exponent (*λ*)-ROS	−3.95	−6.2	More stable but still oscillatory
Lyapunov exponent (*λ*)-immune system	−2.62	−4.8	Adaptive immune response fluctuations
ApEn ROS	0.095	0.02	Higher complexity in chaotic models
ApEn immune system	0.21	0.09	More dynamic and responsive immune oscillations

We can conclude that while ozone therapy is not just an antimicrobial, it restores immune self-organization by modulating chaotic dynamics, so driving immunity to face the pathogenic microbial organisms to promote their clearance. Immune responses are neither rigid nor random, yet they exhibit fractal-like, adaptive oscillations under oxidative stress.

This supports the theory that ozone therapy enhances immune adaptability, improving responses against infections and inflammatory disorders.

The study highlights the potential of ozone therapy as an adjunct treatment against MDR bacteria, emphasizing its role beyond mere antimicrobial action. By employing bioinformatics modelling, the research demonstrates that ozone therapy operates through a combination of direct bactericidal effects and complex immune modulation. The findings suggest that ozone enhances bacterial clearance by inducing controlled oxidative stress, triggering immune system activation and promoting the formation of bioactive mediators such as 4-HNE. This dual mechanism, direct bacterial destruction and immune system modulation, positions ozone as a promising complementary strategy in tackling MDR infections.

However, several limitations must be acknowledged. First, the bioinformatic models used in the study, while valuable for hypothesis generation, remain theoretical and require experimental validation. The study assumes certain decay rates, immune system efficiencies and bacterial responses based on previous literature, but real-world conditions may introduce significant variability. The short half-life of ozone in biological systems further complicates its practical application, necessitating frequent dosage considerations that might not be so impactful in clinical settings. Additionally, while the study explores both linear and chaotic models of ozone action, the actual biological response may be influenced by numerous uncontrolled factors, including individual patient variability, co-infections and underlying health conditions.

Another important limitation is the lack of *in vivo* validation. While ozone has demonstrated antimicrobial properties *in vitro* and in some clinical applications, the precise effects in complex biological environments remain uncertain. The interaction of ozone with host tissues, the potential for oxidative damage and the long-term effects of repeated treatments require further investigation. Moreover, the study does not account for potential adverse effects, such as inflammation or tissue damage due to excessive oxidative stress if ozone dosages are not strictly managed, which could limit the clinical applicability of ozone therapy. Finally, while the study suggests that combining ozone with antibiotics may enhance bacterial clearance, it does not explore potential interactions or resistance mechanisms that bacteria might develop in response to oxidative stress. The possibility that bacteria could adapt to repeated ozone exposure, similar to how they develop antibiotic resistance, remains an open question that warrants further research, although ozone is able to destroy any microbial agent.

Finally, the application of chaos theory and fractal modelling in immunology is increasingly supported by empirical and computational research. These frameworks offer valuable insights into the non-linear, adaptive and dynamic behaviour of immune responses, particularly under stress or infection. Studies have shown that immune cell signalling, cytokine oscillations and the spatial architecture of lymphoid tissues can exhibit fractal patterns and chaotic dynamics. Moreover, measures like Lyapunov exponents and ApEn have been used to quantify immune system complexity in health and disease, including sepsis, autoimmunity and chronic inflammation. While these concepts are not yet part of standard clinical practice, their relevance in systems biology and immuno-informatics is gaining traction. In our manuscript, we employ chaos and fractal theory as modelling tools to conceptualize how ozone therapy might restore adaptive complexity in immune responses. These approaches are intended not as definitive physiological descriptions but as theoretical constructs that align with emerging trends in immunological modelling. We acknowledge the speculative nature of this framework but assert that it is grounded in a legitimate and expanding body of literature. The revised manuscript now clearly frames these concepts as exploratory and includes language to prevent overinterpretation, ensuring a balanced and scientifically transparent discussion [32,33].

While foundational concepts were developed in prior publications by the authors, future versions of this work should incorporate a broader range of independent, peer-reviewed literature to enhance external validation and credibility.

In conclusion, while the findings support the potential of ozone therapy as a tool against MDR bacteria, its clinical translation requires careful validation through rigorous experimental and clinical studies. The limitations identified highlight the need for further research to refine dosing strategies, assess long-term safety and determine the most effective protocols for integrating ozone therapy into existing antimicrobial treatments.

## Limitations of the study

This study is grounded entirely in theoretical modelling and bioinformatic simulations, with no accompanying experimental or clinical data to validate the proposed frameworks. All models presented, whether based on linear decay kinetics or adaptive chaos, are hypothetical constructs designed to generate hypotheses rather than to offer definitive clinical or biological conclusions. Consequently, the outcomes should be interpreted as illustrative rather than predictive.

Several concepts discussed, such as ‘adaptive chaos’, ‘immune system resilience’ and ‘fractal immune responses’, while novel and conceptually stimulating, currently lack robust empirical validation in the context of immunology or infectious disease treatment. The application of complex systems theory, including chaotic attractors and entropy-based measures, to biological immune responses is speculative. These models attempt to capture the non-linear and dynamic nature of biological systems but should not be interpreted as representations of established physiological mechanisms without supporting experimental evidence.

Furthermore, the absence of *in vitro*, *in vivo* or clinical data significantly limits the translational potential of the findings. Critical biological parameters, such as immune system responsiveness, microbial adaptation and interindividual variability, were simplified or inferred from previous literature. Without empirical grounding, these assumptions may not accurately reflect real-world biological behaviour, of course, especially in a clinical setting involving MDR bacterial infections. Anyway, numerous peer-reviewed studies have demonstrated the antimicrobial efficacy of ozone, including its action against MDR bacteria [16,18, 34,37]. These studies, conducted both *in vitro* and *in vivo*, have shown that ozone can significantly reduce bacterial loads of strains such as MRSA, *P. aeruginosa* and *A. baumannii*, even when traditional antibiotics fail. Clinical trials and experimental models have confirmed ozone bactericidal potential via oxidative mechanisms that disrupt bacterial membranes, proteins and genetic material [16,18, 34,37]. This growing body of evidence supports the rationale for exploring ozone as an adjunct therapeutic strategy in infections caused by resistant pathogens, complementing the modelling presented in this article.

We acknowledge that while the modelling approach introduces a potentially insightful perspective, its scientific impact is contingent upon future empirical studies. These should aim to validate the model assumptions, quantify key biological parameters and test the efficacy and safety of ozone therapy under controlled conditions. Until such studies are conducted, the current work should be viewed as a theoretical exploration rather than a basis for clinical application.

## Ozone safety profile

The therapeutic use of ozone, as discussed in this study, is strictly confined to the hormetic range (typically 20–80 µg ml^−1^), within which ozone has consistently been shown to be non-toxic and well-tolerated. The hormetic range represents a threshold where low doses of an otherwise potentially harmful agent trigger beneficial biological responses without causing damage. In the case of ozone, such dosing activates controlled oxidative signalling pathways that stimulate antioxidant defences, modulate immune function and promote homeostatic repair processes. Numerous clinical and preclinical studies have confirmed that within this range, ozone does not cause inflammatory or cytotoxic effects. In fact, when administered correctly, it enhances systemic resilience by reinforcing redox balance rather than disrupting it. In contrast, adverse effects such as oxidative tissue damage, inflammation or mitochondrial dysfunction are primarily associated with ozone exposure at high concentrations or through inappropriate routes (e.g. direct inhalation), which are categorically excluded from therapeutic protocols. The established medical use of ozone involves strictly regulated concentrations and delivery methods (e.g. major autohaemotherapy, rectal insufflation) that avoid such risks. Additionally, clinical practitioners must adhere to standardized protocols, such as those recommended by professional societies (e.g. Italian Scientific Society of Oxygen-Ozone Therapy, SIOOT, Gorle, BG, Italy), to ensure both efficacy and safety. In conclusion, while we recognize the importance of vigilance regarding any pro-oxidative intervention, current evidence supports the safety of ozone therapy within the defined hormetic window. The manuscript now reflects this nuanced understanding, providing a critical and evidence-based evaluation of both therapeutic benefits and safety boundaries.

## Conclusions

This study presents a theoretical framework using bioinformatic modelling to evaluate ozone therapy as an adjunct treatment for MDR infections. Through linear and non-linear simulations, including adaptive chaos models, the research suggests that ozone may enhance bacterial clearance by combining direct oxidative effects with complex immune modulation. Central to this hypothesis is the hormetic application of ozone, which may induce the production of immunomodulatory molecules such as 4-HNE and stimulate a self-regulating, dynamic immune response. The findings highlight ozone potential to restore immune complexity and adaptability, beyond its antimicrobial action. However, these outcomes are speculative and based solely on theoretical constructs; no *in vitro* or clinical data were presented. The models should therefore be interpreted as hypothesis-generating tools rather than predictive clinical strategies. Experimental studies are urgently needed to validate the proposed mechanisms, quantify dosing responses and evaluate long-term safety and efficacy in real-world clinical settings.

Future work should include quantitative sensitivity analyses to assess how variation in key parameters, such as immune response efficiency, ozone decay rates and 4-HNE kinetics, affects model predictions. This would enhance the robustness and translational relevance of the simulations.

## Abbreviations

ApEn: approximate entropy
4-HNE: 4-hydroxynonenal
LPO: lipoperoxide
MDR: multidrug-resistant
MRSA: methicillin-resistant *Staphylococcus aureus*
NSAIDs: non-steroidal anti-inflammatory drugs
ODEs: ordinary differential equations
ROS: reactive oxygen species
WHO: World Health Organization
